# Identifying the most suitable endogenous control for determining gene expression in hearts from organ donors

**DOI:** 10.1186/1471-2199-8-114

**Published:** 2007-12-20

**Authors:** Silvia Pérez, Luis J Royo, Aurora Astudillo, Dolores Escudero, Francisco Álvarez, Aida Rodríguez, Enrique Gómez, Jesús Otero

**Affiliations:** 1 Unidad de Coordinación de Trasplantes y Terapia Celular, Hospital Universitario Central de Asturias, C/Celestino Villamil s/n, 33006 Oviedo, Spain; 2 Área de Genética y Reproducción Animal, SERIDA-Somió, C/Camino de los Claveles 604, 33203 Gijón, Spain; 3 Servicio de Anatomía Patológica, Hospital Universitario Central de Asturias, Oviedo, Spain; 4 Servicio de Medicina Intensiva, Hospital Universitario Central de Asturias, Oviedo, Spain; 5 Servicio de Bioquímica Clínica, Hospital Universitario Central de Asturias, Oviedo, Spain

## Abstract

**Background:**

Quantitative real-time reverse transcription PCR (qRT-PCR) is a useful tool for assessing gene expression in different tissues, but the choice of adequate controls is critical to normalise the results, thereby avoiding differences and maximizing sensitivity and accuracy. So far, many genes have been used as a single reference gene, without having previously verified their value as controls. This practice can lead to incorrect conclusions and recent evidence indicates a need to use the geometric mean of data from several control genes. Here, we identified an appropriate set of genes to be used as an endogenous reference for quantifying gene expression in human heart tissue.

**Results:**

Our findings indicate that out of ten commonly used reference genes (*GADPH, PPIA, ACTB, YWHAZ, RRN18S, B2M, UBC, TBP, RPLP and HPRT*), *PPIA*, *RPLP *and *GADPH *show the most stable gene transcription levels in left ventricle specimens obtained from organ donors, as assessed using geNorm and Normfinder software. The expression of *TBP *was found to be highly regulated.

**Conclusion:**

We propose the use of *PPIA*, *RPLP *and *GADPH *as reference genes for the accurate normalisation of qRT-PCR performed on heart tissue. *TBP *should not be used as a control in this type of tissue.

## Background

Gene expression analysis is a useful technique for determining and comparing gene expression levels in healthy and diseased tissues. One of the most commonly used tools in the area of gene expression quantification is quantitative real-time reverse transcription PCR (qRT-PCR). When small amounts of nucleic acids are available, qRT-PCR is especially suitable and provides simultaneous measurement of gene expression in many different samples. If we compare this technique with others such as in situ hybridisation, qRT-PCR offers several advantages: it is not time-consuming, only a small amount of tissue is required, it can be used in high throughput systems and no post-reaction manipulation is needed. However, the use of qRT-PCR requires compensation for differences between samples, arising from the varying quality and quantity of the starting material, especially when starting with solid tissue, due to the method of RNA extraction and cDNA synthesis [1]. Normalisation should include endogenous control genes (reference genes), and some of the most frequently used reference genes are housekeeping genes. The ideal endogenous control should be expressed at a constant level in the different tissues of an organism at all stages of development and should be unaffected by experimental treatments. It should also be constitutively expressed in the same tissue under different circumstances. There is, however, no universal control gene that is expressed at a constant level under all conditions and in all tissues. Hence, experimental treatments [2] and hormonal stimulation [3], as well as the different methods used to process tissue specimens [4] can induce changes in the expression of typical housekeeping genes.

Consequently, the success of the technique used depends on the adequate choice of the appropriate reference genes. Despite many qRT-PCR studies having reported the use of a single endogenous control [5], a normalisation strategy based on a single housekeeping gene can lead to erroneous results [6,7]. Vandesompele *et al*. [6] propose the use of a panel of putative reference genes on a representative number of samples, to identify the most stable of these and then establish the optimal number of genes required for the reliable normalisation of RT-PCR data. In the present study, we tested ten commonly used reference genes (*GADPH, PPIA, ACTB, YWHAZ, RRN18S, B2M, UBC, TBP, RPLP and HPRT*) of different functional classes (significantly reducing the chance that the genes will be co-regulated) in heart tissue obtained from organ donors. Using geNorm software [6], we were able to assess gene expression stability under our experimental conditions and determine how many reference genes were needed for accurate normalisation. Then, by comparing these results with those generated by a similar programme, Normfinder [8,9], we identified a set of reference genes that offers reliable results for qRT-PCR data normalisation for use in gene expression studies involving heart tissue from brain-dead multiorgan donors.

## Results

Thirty five samples of left ventricular tissue were obtained from 35 multiorgan donors. RNA was successfully isolated and cDNA synthesised from all these specimens. All the samples analysed showed a single *β-actin *band in the 2% agarose gel stained with ethidium bromide at the expected size (data not shown), confirming the total absence of residual DNA.

**Figure 1:**
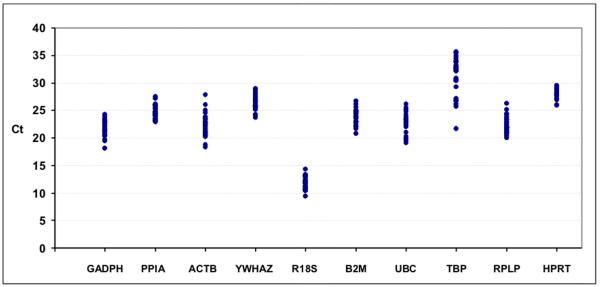
**Expression levels of candidate housekeeping genes**. Scatter plots showing the expression levels of the different reference genes in the tested heart samples (n = 35). Values are given as cycle threshold numbers (Ct values).

In each sample, qRT-PCR using Sybr Green was performed for ten frequently-used reference genes (*GADPH, PPIA, ACTB, YWHAZ, RRN18S, B2M, UBC, TBP, RPLP and HPRT*). The accuracy of the qRT-PCR was assessed by melt curve analysis and gel electrophoresis. Gene-specific amplification was verified, by both a single peak in the melt curve and a single band in the agarose gel, for the 10 genes analysed in the 35 cDNA samples. Correlation coefficients (R^2^) ranged from 0.995 to 0.999 and PCR efficiencies from 89.7% to 104%. Using the Proc VARCOMP in the SAS/STAT™ software [10], the reproducibility of the assay was assessed using as control material samples obtained by pooling the whole set of samples analysed in the present study. The high average correlation coefficient observed of 0.998 indicated good intra-sample reproducibility. Observed Ct values for each gene were similar across different samples indicating low variability (21.66 ± 1.39 for GADPH, 24.69 ± 1.22 for PPIA, 22.32 ± 2.03 for ACTB, 26.39 ± 1.36 for YWHAZ, 11.71 ± 1.10 for RRN18S, 23.87 ± 1.37 for B2M, 22.71 ± 1.88 for UBC, 30.98 ± 3.45 for TBP, 22.27 ± 1.42 for RPLP and 28.08 ± 0.94 for HPRT) (Figure 1).

**Figure 2:**
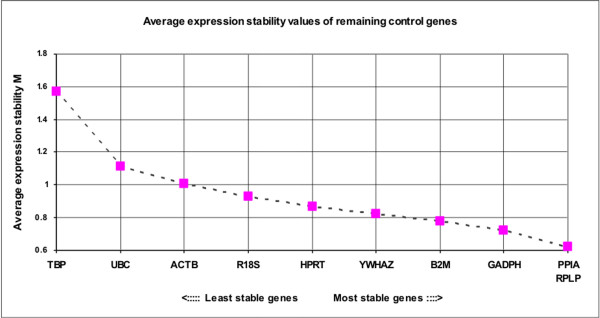
**Average expression stability values (M) of the candidate reference genes**. Average expression stability measure (M) of control genes during stepwise exclusion of least stable reference genes. M is represented from the least stable (left) to the most stable (right), analysed by the geNorm programme.

**Figure 3:**
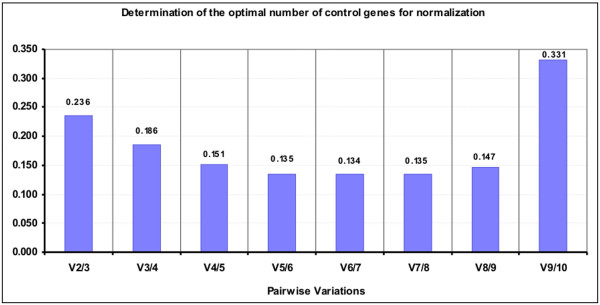
**Pairwise variation analysis between normalisation factors to determine the optimal number of control genes for normalisation**. Optimal number of control genes for normalisation calculated on the basis of pairwise variation (V) analysis. The highest V9/10 value is due to the inclusion of a relative unstable gene and is in accordance with the average expression stability (M).

The GeNorm software also provided a rank order of the ten candidate reference genes according to their M values (Figure 2). *PPIA*, *RPLP *and *GADPH *were the three most stable genes. M values increased moderately for all genes, while *TBP *abruptly attained the highest M value. The pairwise variation (V) upon normalisation with the two most stable genes and introduction of the third one was 0.236. This value decreased gradually until the addition of the fifth gene, when the trend became more or less stable but increased considerably upon the incorporation of the tenth gene (Figure 3).

Table 1 provides the results of the Normfinder analysis performed on our data. The reference genes tested were ordered according to their stability values. Thus, the most stable single gene was GADPH with a stability value of 0.345 and the least stable was TBP with a value of 2.287.

**Table 1:** Tested reference genes for normalisation of qRT-PCR listed according to their expression stability calculated by the Normfinder software.

**Raking Order**	**Gene Name**	**Stability Value**
1	GADPH	0.345
2	PPIA	0.405
3	RPLP	0.426
4	B2M	0.446
5	YWHAZ	0.498
6	HPRT	0.588
7	RRN18S	0.593
8	ACTB	0.737
9	UBC	0.831
10	TBP	2.287

## Discussion

Human heart tissue specimens are very difficult to acquire. However, we managed to obtain 35 samples from the left ventricles of 35 organ donors of different age, weights and sex, whose hearts could not be used for transplant purposes for various reasons (see Methods). Thus, we can assume that the number and type of samples used was sufficiently high and diverse to ensure their representativity and randomness. Reports of the use of qRT-PCR in human heart tissue are scarce because of the technical difficulties involved in obtaining such samples. However, given that qRT-PCR is especially suitable for determining gene expression in small pieces of tissue, we tried to establish guidelines for accurate data normalisation intended for human heart studies based on qRT-PCR.

Our technical procedure proved to be a valid method of quantifying gene expression, rendering high correlation coefficients (R^2^) and robust PCR efficiencies.

Through geNorm analysis, *PPIA*, *RPLP *and *GADPH *appeared as the most stable genes and *TBP *the least stable (Figure 2). It has been reported that in over 90% of cases, gene expression data are normalised using *GADPH*, *ACTB*, *18S rRNA *or *28S rRNA *as single control genes [5]. However, several studies have shown that these reference genes undergo variation according to the experimental conditions, treatment and cell cycle stage [11,12].

In human adipocytes and preadipocytes, *ACTB *and *18S rRNA *gene expression levels change under hormonal stimulation [3]. Moreover, it has been reported that *ACTB *is unsuitable as a control for gene expression analysis in interstitial cells derived from sheep heart valves [13]. The *18S rRNA *gene has been considered an ideal internal control in qRT-PCR analysis. However, ribosomal RNA accounts for up to 80–90% of total cellular RNA, and several studies have shown that rRNA varies less under conditions that affect the expression of mRNAs [14] but that possible imbalances in rRNA and mRNA fractions between different samples makes genes encoding ribosomal RNAs unsuitable as references [6]. As far as we are aware, no previous study has tried to identify adequate housekeeping genes for use in human heart tissue. Morgan *et al*. [15] examined the expression of genes related to the renin-angiotensin system in human atrial tissue using *GADPH *as an endogenous control. Other authors [16] have used RNA extracted from human endomyocardial biopsies and isolated cardiomyocytes for real-time RT-PCR using *GADPH*, *HPRT *and the oncogene *ABL *as housekeeping genes. These endogenous controls showed very low variation in individual gene expression levels across cardiac pathologies, suggesting their suitable use as reference genes for quantitative PCR studies in cardiac tissue. In these previous studies, the specific testing of several candidate reference genes to determine the most suitable reference for use in cardiac tissue was not reported [15,16]. In contrast, Radonic et al. [17] determined transcription levels of several housekeeping genes in different human tissues, including heart, and identified TBP as the gene with the lowest range of RNA transcription across tissues. This finding is in agreement with the present results.

We found *PPIA*, *RPLP *plus *GADPH *to be a reliable set of genes for normalising data (Figure 3) according to the geNorm programme. As reported by Vandesompele *et al*. [6], geNorm proposes a pairwise variation of 0.15 as the cut-off under which the inclusion of an additional control gene is not required. Using our set of candidate genes, this would mean that adding a fifth gene to the four most stable genes identified would really provide the best results. Notwithstanding, this cut-off of 0.15 should not be considered in a strict sense, but rather as a reference to determine the optimal number of housekeeping genes. Sometimes the observed trend can be equally informative, and using the three best reference genes is, in most cases, a valid strategy for much more accurate and reliable normalisation compared to the use of a single housekeeping gene.

Using NormFinder software [8] as another tool to validate the expression stability of the ten candidate reference genes; GADPH, PPIA and RPLP also achieved the best stability values. While geNorm detected the two genes whose expression ratios showed least variation from those of the other genes tested, NormFinder was able to identify the single gene with the most stable expression. Hence, the most stable candidate gene was found to be GADPH, and the least stable TBP. Using this programme, we obtained the same results as with GeNorm except for the rank position ascribed to GADPH as the most stable single gene. However, the least stable genes and most stable ones identified by GeNorm and Normfinder were generally well-matched.

As a limitation to our study, we should mention that although we established the purity of our RNA samples, due to the amount of RNA available, we could not run electrophoretic tests to check RNA integrity.

Finally, the set of reference genes determined here as the best endogenous controls to be used in qRT-PCR studies in heart tissue has applications in developing cardiovascular diagnostic tests and therapeutic strategies that will substantially improve human health [18].

## Conclusion

It is commonly accepted that gene expression studies should be normalised using more than one reference gene. Based on our results, we propose the use of the mean result rendered by *PPIA*, *RPLP *and *GADPH *as housekeeping genes to normalise gene expression values obtained by qRT-PCR in heart tissue.

## Methods

### Sample collection

Tissue samples were obtained from the left ventricle of 35 organ donors (19 male, 16 female; mean age 59 years, range 40 – 80 years). The organ donors had died in a multidisciplinary ICU of a tertiary university hospital. In 85% of the organ donors, brain death was attributed to a non-traumatic cause and no donor had previous heart pathology. Consent for organ donation for transplant and clinical investigation was obtained from the relatives of each donor.

The hearts had been rejected for transplant because of donor age or weight, or recipient blood group incompatibility. The cardiac tissues were freshly harvested, stored immediately in O.C.T™ Compound (Tissue Tek, Sakura, Netherlands) and then frozen at -80°C.

### RNA extraction and cDNA synthesis

Isolation of total RNA was performed using the Nucleo Spin RNA II Isolation Kit (Macherey-Nagel Gmb H & Co. KG) according to the manufacturer's instructions.

The quality of the RNA was assessed through absorbance measurements made using a NanoDrop ND-1000 UV-Vis Spectrophotometer (NanoDropTechnologies, Wilmington, DE, USA). All the samples used in the PCR procedures showed a 260/280 nm absorbance ratio between 1.8 and 2.2. A ratio of ≈2 is generally accepted as pure for RNA.

Each RNA sample was reverse transcribed using the Oligo-pdT and Random primers provided in the first-strand complementary DNA (cDNA) synthesis kit for RT-PCR (AMV; Roche Diagnostic, Switzerland) following the manufacturer's instructions. The tubes were incubated at 25°C for 10 minutes to allow annealing. Reverse transcription was conducted at 42°C for 60 minutes, followed by 5 minutes incubation at 99°C to denature the enzyme. The cDNA samples were then cooled at 4°C and stored at -20°C until use.

To confirm the total absence of residual DNA, we performed a conventional PCR with *β-actin *[GenBank:E00829] primers designed using Beacon Designer software (Premier Biosoft International, California) spanning intron-exon boundaries (*β-actin *up 5'-GACTACCTCATGAAGATCCTC-3'; *β-actin *down 5'-CGGATGTCCACGTCACACTTC-3'). The PCR protocol consisted of an initial denaturation step at 94°C for 5 minutes, followed by 35 amplification cycles. Each cycle involved a denaturation step of 30 seconds at 94°C, followed by 30 seconds of primer annealing at 55°C and 30 seconds of primer extension at 72°C followed by a final extension of 10 minutes. Amplicon size was confirmed by electrophoresis using an ethidium bromide-stained 2% agarose gel in 1XTBE buffer.

### Gene expression

We analysed the expression of the genes *GADPH, PPIA, ACTB, YWHAZ, RRN18S, B2M, UBC, TBP, RPLP *and *HPRT *included in the Human Endogenous Control Gene Panel supplied by TATAA Biocenter (Goteborg, Sweden) (see Table 2). For this panel, the genes are carefully selected to be of different functional classes, thus significantly reducing the likelihood that they might be co-regulated. The genes selected here were also evaluated by Vandesompele *et al*. [6].

**Table 2:** Abbreviations and functions of the genes mentioned in the text

**GADPH**	Glyceraldehyde-3-phosphate dehydrogenase	Glycolytic enzyme
**PPIA**	Peptidylprolyl isomerase A (cyclophilin A)	Immunity protein
**ACTB**	Actin beta	Cytoskeletal structural protein
**YWHAZ**	Tyrosine 3/tryptophan 5-monooxygenase activation protein, zeta polypeptide	Protein signal transduction
**RRN18S**	18S rRNA	Ribosomal RNA
**B2M**	Beta-2-microglobulin	Beta-chain of major histocompatibility complex class I molecules
**UBC**	Ubiquitin C	Protein degradation
**TBP**	TATAA box binding protein	General RNA polymerase II transcription factor
**RPLP**	60S Acidic ribosomal protein P0	Member of the ribosome proteins
**HPRT**	Hypoxanthine phosphoribosyltransferase	Purine synthesis

Quantitative PCR was performed using an i-Cycler iQ Real-Time PCR Detection System (Bio-Rad, Hercules, CA, USA). SYBR Green PCR Supermix (2X) (Bio-Rad), which detects amplification using Sybr Green as a double-stranded DNA-specific fluorescent dye, was used as a mastermix. Assays were performed in duplicate and a blank included in every assay. The reaction mixture for amplification consisted of 2 μl of cDNA in a final reaction volume of 25 μl, as indicated in the instructions for the Human Endogenous Control Gene Panel. The RT-PCR protocol included an initial step of 95°C (3 minutes), followed by 40 cycles of 30 seconds at 95°C for DNA denaturation, 20 seconds for primer annealing at 60°C and 20 seconds at 72°C for primer extension. Fluorescence data were acquired at 72°C. Melting-curve analysis to confirm product specificity was performed immediately after amplification, following 1 minute denaturation at 95°C, 1 minute annealing at 65°C and 60 cycles of 0.5°C increments (10 seconds each) beginning at 65°C while monitoring fluorescence. Product identity was confirmed by electrophoresis using an ethidium bromide-stained 2% agarose gel in 1XTBE buffer.

PCR efficiencies were calculated from a relative standard curve derived from a pool of heart tissue specimens from the same organ donors and serially diluted (a ten-fold dilution series with at least six measuring points).

### Reference gene expression stability

To determine the most stable housekeeping genes of the set of tested genes in our heart cDNA sample panel, we used firstable, the geNorm software package for Microsoft Excel^® ^developed by Vandesompele *et al*. [6]. The programme geNorm uses an algorithm to calculate M, a gene expression stability measure, defined as the mean pairwise variation for a given gene compared to the remaining tested control genes. Thus, the higher the M value of a gene, the greater the variation in its expression levels. The programme establishes a rank order of gene stability via stepwise exclusion. To determine how many reference genes should be used, normalisation factors (NF_n_) based on the geometric mean of the expression levels of the n best reference genes were calculated by stepwise inclusion of an extra, less stable reference gene as described elsewhere [4]. The programme shows the pairwise variation Vn/V_n+1 _between two sequential normalisation factors, NFn and NF_n+1_. A large variation means that the added gene has a significant effect and should probably be included for calculation of the normalisation factor.

To compare our results with those generated by a similar programme, we used Normfinder [9], another algorithm-based tool for identifying optimal reference genes among a set of candidates. The programme ranks the genes depending on their expression stability value in given samples derived from an experimental design.

## Authors' contributions

The first author of the manuscript SP performed the experimental procedures. LJR, AR and EG provided support for the qRT-PCR and discussion. AA and FA harvested the heart tissue. DE was the main person responsible for obtaining tissue samples from organ donors. The project was designed by JO. All the authors have read and approved the final manuscript.
